# Vaccination Efficacy of Mitoxantrone-Treated CT-26 Cells Under Different Drug Concentrations and Vaccination Intervals

**DOI:** 10.3390/molecules31213827

**Published:** 2026-10-23

**Authors:** Se Won Na, Mibae Jeong, Mi-Kyung Jeong, Jaemoo Chun

**Affiliations:** 1 KM Convergence Research Division, Korea Institute of Oriental Medicine, Daejeon 34054, Republic of Koreaoiny2000@kiom.re.kr (M.-K.J.); 2 Department of Pharmaceutical Engineering, Gyeongsang National University, Jinju 52828, Republic of Korea; 3 Department of Science in Korean Medicine, College of Korean Medicine, Kyung Hee University, Seoul 02447, Republic of Korea; 4 KIOM School, University of Science & Technology (UST), Daejeon 34054, Republic of Korea

**Keywords:** mitoxantrone, CT-26, colon carcinoma, immunogenic cell death, prophylactic vaccination

## Abstract

Immunogenic cell death (ICD) converts dying tumor cells into an anticancer vaccine, yet whether protection depends on how those cells are prepared and when they are given has rarely been examined. Using mitoxantrone (MTX), a clinically approved small molecule classified as an ICD inducer, we examined both variables in the murine CT-26 colon carcinoma model. MTX reduced viability in a concentration-dependent manner and induced extracellular ATP release. The total extent of cell death, however, was similar across concentrations, indicating that the concentration-dependent loss of viability was not accompanied by a proportional increase in cell death. What differed was the composition of the dying population, which shifted from early to late apoptotic cells as the concentration increased. Prophylactic vaccination with MTX-treated cells protected BALB/c mice, leaving 75.0–87.5% of animals tumor-free after vaccination at 1 μM and 37.5–62.5% at 3 μM across independent experiments. Protection increased with the interval between vaccination and challenge, with 0%, 50.0%, and 87.5% of animals tumor-free after simultaneous injection, a 3-day interval, and a 7-day interval, respectively. Cells killed completely by freeze–thaw were inactive as a vaccine, and cisplatin-treated cells conferred only a marginal delay, whereas responders to MTX-treated cells resisted rechallenge for the remainder of the observation period. Taken together, these results show that use of an ICD-inducing drug alone is not sufficient for effective dying-cell vaccination and that protective efficacy is influenced by vaccine preparation and by the interval between vaccination and tumor challenge, rather than by the extent of cell death alone.

## 1. Introduction

Immunogenic cell death (ICD) is a functionally defined form of regulated cell death in which dying tumor cells acquire adjuvant properties and prime an adaptive antitumor response [1]. The concept has moved from a mechanistic observation to a therapeutic principle, since agents that elicit ICD convert immunologically cold tumors into inflamed lesions, and their combination with immune checkpoint blockade is now among the most actively pursued strategies for extending the benefit of immunotherapy to poorly responsive tumors [2,3]. Immunogenicity here reflects two separable properties of a dying cell, which are antigenicity, set by the epitopes it carries, and adjuvanticity, set by the signals it emits as it dies [4]. While the intrinsic antigenic potential of a given cell line is defined by its baseline epitope repertoire, experimental studies of ICD frequently focus on manipulating adjuvanticity, which is mediated by damage-associated molecular patterns (DAMPs) sensed by antigen-presenting cells. The three canonical DAMPs are emitted sequentially rather than together. Calreticulin reaches the cell surface before apoptosis becomes overt and acts as an eat-me signal [5]. ATP is secreted during early apoptosis as a find-me signal and inflammasome activator [6]. HMGB1 is released predominantly after loss of plasma membrane integrity [7,8]. A preparation captured at one point along this trajectory therefore presents different signals from one captured later.

That cell death is regulated does not make it immunogenic. Apoptotic cells are ordinarily cleared by efferocytosis without inflammation, and this clearance actively promotes tolerance rather than priming [4]. Accidental necrosis, in which intracellular contents are released without any preceding stress response, is likewise a poor immunogen despite the abundance of material released. Necroptosis and pyroptosis are generally reported to be immunogenic [9], whereas ferroptotic cells have been found either to protect against subsequent challenge or to impair dendritic cell function, depending on the stage of death at which they were administered [10,11]. For this reason, ICD cannot be diagnosed from DAMP measurements alone and must be established functionally by prophylactic vaccination in which immunocompetent mice are inoculated with dying tumor cells and subsequently challenged with live cells of the same line [12,13]. Mitoxantrone (MTX), a synthetic anthracenedione that intercalates into DNA and acts as a topoisomerase II poison, is approved for several hematologic and solid malignancies [14] and is a commonly used reference compound in experimental studies of ICD. Its immunogenicity depends on a pre-apoptotic endoplasmic reticulum stress response, in which PERK-mediated phosphorylation of eIF2α precedes anterograde transport of calreticulin to the cell surface. Cytotoxic agents that kill efficiently without engaging this pathway, cisplatin among them, fail to elicit protection, and their immunogenicity can be restored by pharmacological induction of ER stress [15,16]. The distinction between the two is therefore mechanistic rather than a matter of potency.

Despite this widespread use, the conditions under which MTX-treated cells confer protection have rarely been examined systematically. Most studies use a single concentration and a fixed vaccination-to-challenge interval [17,18]. Where the conditions of killing have been varied deliberately, they have proved to matter. In the same CT-26 model, the immunogenicity of photodynamic therapy depends on both photosensitizer concentration and light dose, and milder protocols outperform more intense ones [19]. In ferroptosis, the stage reached before injection determines whether dendritic cells are engaged productively at all [11]. The vaccination-to-challenge interval is also often fixed, typically at approximately one week, limiting direct assessment of its contribution to vaccine efficacy. More fundamentally, the composition of the injected material is not consistently reported, even though the DAMPs that define ICD are emitted at distinct stages of the death process. Cells exposed to the same compound at different concentrations may thus differ in the proportion of viable, early apoptotic, and late apoptotic cells they contain and present different signals to the immune system while being described in identical terms. Incomplete reporting of experimental conditions can make comparisons across preclinical studies difficult [20].

We addressed these questions in the CT-26 model. Protection in this model depends substantially on the endogenous gp70-derived immunodominant epitope AH1 [21], whose deletion abolishes the protective effect of apoptotic cell vaccination [22]. Among the syngeneic mouse tumors in common use, CT-26 is one of the most immunogenic and the most responsive to checkpoint blockade [23], providing a model in which vaccination can be readily evaluated and in which the effects of experimental conditions can be examined. If the result varies with the way the vaccine is prepared even where a strong immunodominant antigen is available, the same variables are likely to be more important in models where priming is more difficult.

Here we used MTX as a reference compound to examine these variables. We first defined the concentration–response relationship and then characterized the death profile and DAMP output of cells prepared under conditions matching those used for vaccination. We next varied two parameters that are usually held fixed, the concentration used to generate the dying-cell vaccine and the interval between vaccination and challenge, and benchmarked MTX against a non-immunogenic form of killing (freeze–thaw) and a cytotoxic agent that does not engage the ICD program (cisplatin). Finally, we assessed the durability of the resulting immunity by rechallenge. Our results show that the extent of cell death is a poor predictor of vaccination efficacy and that the state of the inoculum and the timing of its administration govern the outcome.

## 2. Results

### 2.1. MTX Reduces CT-26 Viability in a Concentration-Dependent Manner

**Figure 1:**
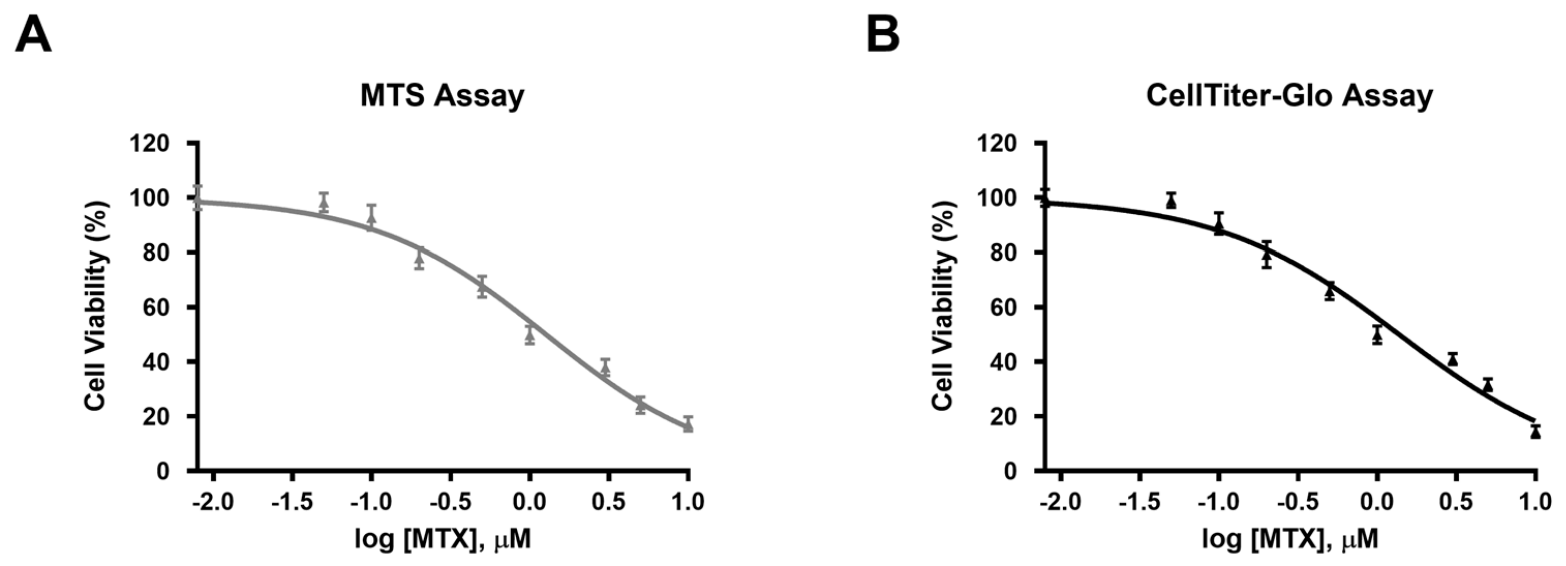
Mitoxantrone (MTX) reduces CT-26 viability in a concentration-dependent manner. CT-26 cells were seeded at 1 × 10^4^ per well in 96-well plates, exposed to MTX (0.05–10 μM) or vehicle for 24 h, and viability was assessed by (**A**) MTS assay and (**B**) CellTiter-Glo assay. Viability is expressed relative to vehicle-treated controls. Data are the mean ± SD of three independent experiments (*n* = 3). Curves are four-parameter logistic fits with the bottom and top constrained to 0 and 100%.

To define the working range for vaccine preparation, CT-26 cells were exposed to MTX (0.05–10 μM) or vehicle for 24 h, and viability was assessed by MTS, which reports mitochondrial dehydrogenase activity, and CellTiter-Glo, which quantifies intracellular ATP. MTX reduced viability in a concentration-dependent manner, with a half-maximal inhibitory concentration of 1.27 μM by MTS (Figure 1A) and 1.38 μM by CellTiter-Glo (Figure 1B). Although the two assays read out different cellular parameters, they gave closely similar values.

### 2.2. MTX Causes a Concentration-Dependent Shift in the Death Profile of Treated Cells

**Figure 2:**
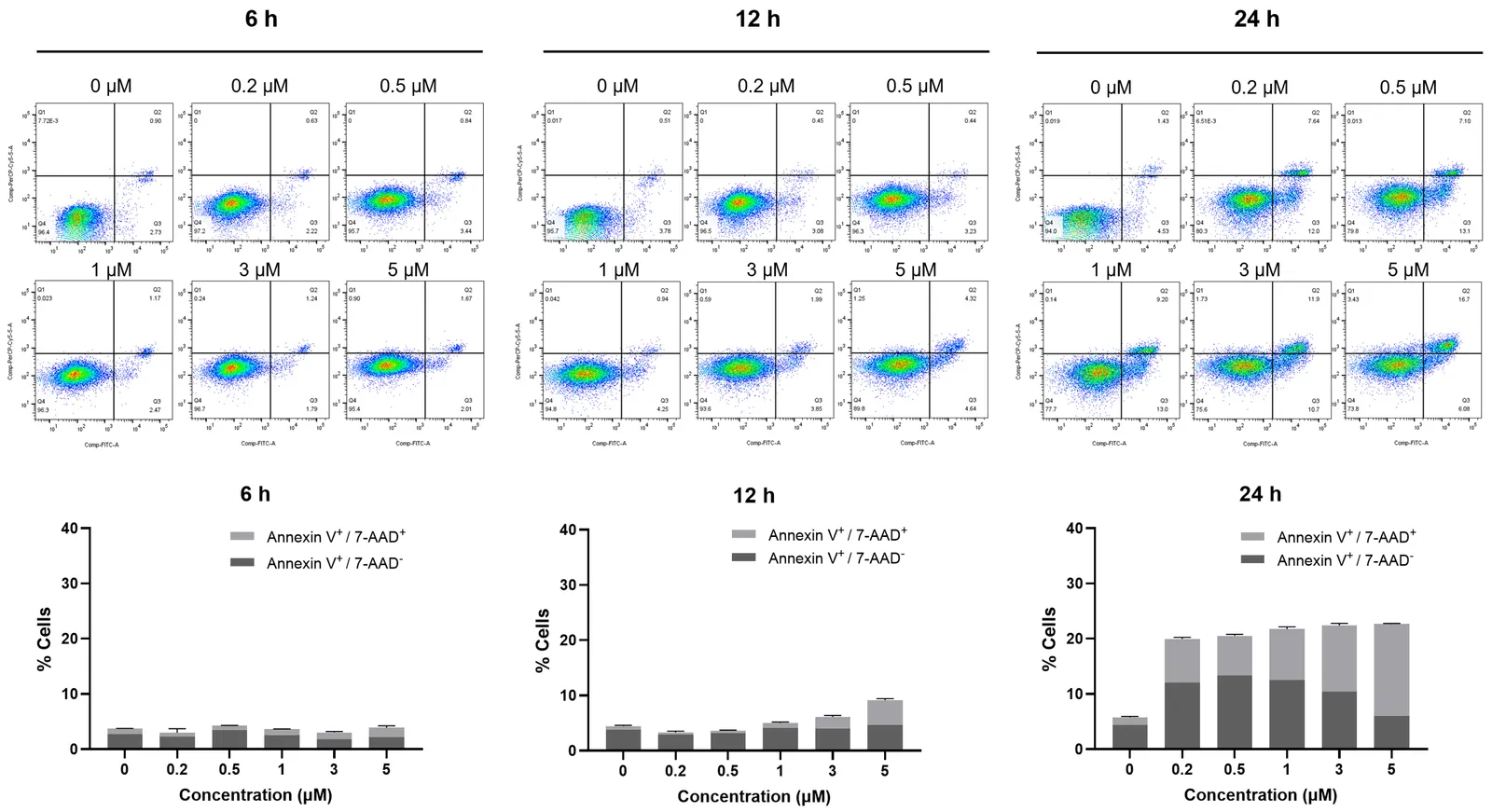
MTX causes a concentration-dependent shift in the death profile of CT-26 cells. Cells were exposed to MTX (0.2–5 μM) or vehicle for 6, 12, or 24 h and stained with Annexin V and 7-AAD. Upper panels show representative flow cytometry plots at each time point and concentration. The horizontal axis (FITC) corresponds to Annexin V and the vertical axis (PerCP-Cy5.5) to 7-AAD. Lower panels show the proportion of Annexin V^+^/7-AAD^−^ (early apoptotic) and Annexin V^+^/7-AAD^+^ (late apoptotic) cells. Data are from a representative experiment and are shown as mean ± SD of three technical replicates (*n* = 3).

To determine how much of the loss of viability reflected cell death and in what form, CT-26 cells were exposed to MTX (0.2–5 μM) or vehicle for 6, 12, or 24 h and stained with Annexin V and 7-AAD (Figure 2). Cell death was minimal at 6 h and remained close to control levels at all concentrations. By 12 h, a modest increase was apparent only at the higher concentrations (3 and 5 μM). At 24 h, Annexin V^+^ cells accounted for 20–23% of the population at all MTX concentrations tested. The proportion of dying cells did not scale with the loss of viability measured over a comparable concentration range, suggesting that the concentration-dependent loss of metabolic viability was not accompanied by a proportional increase in overt cell death. Although the overall proportion of Annexin V-positive cells at 24 h was similar across concentrations, the composition of this population differed markedly. Early apoptotic cells (Annexin V^+^/7-AAD^−^) predominated at concentrations up to 1 μM, whereas late apoptotic cells (Annexin V^+^/7-AAD^+^) predominated at 3 and 5 μM. Thus, increasing the concentration did not increase the amount of cell death at this time point but advanced the population further along the death process.

### 2.3. MTX Induces Extracellular ATP Release with Concentration-Dependent Kinetics

**Figure 3:**
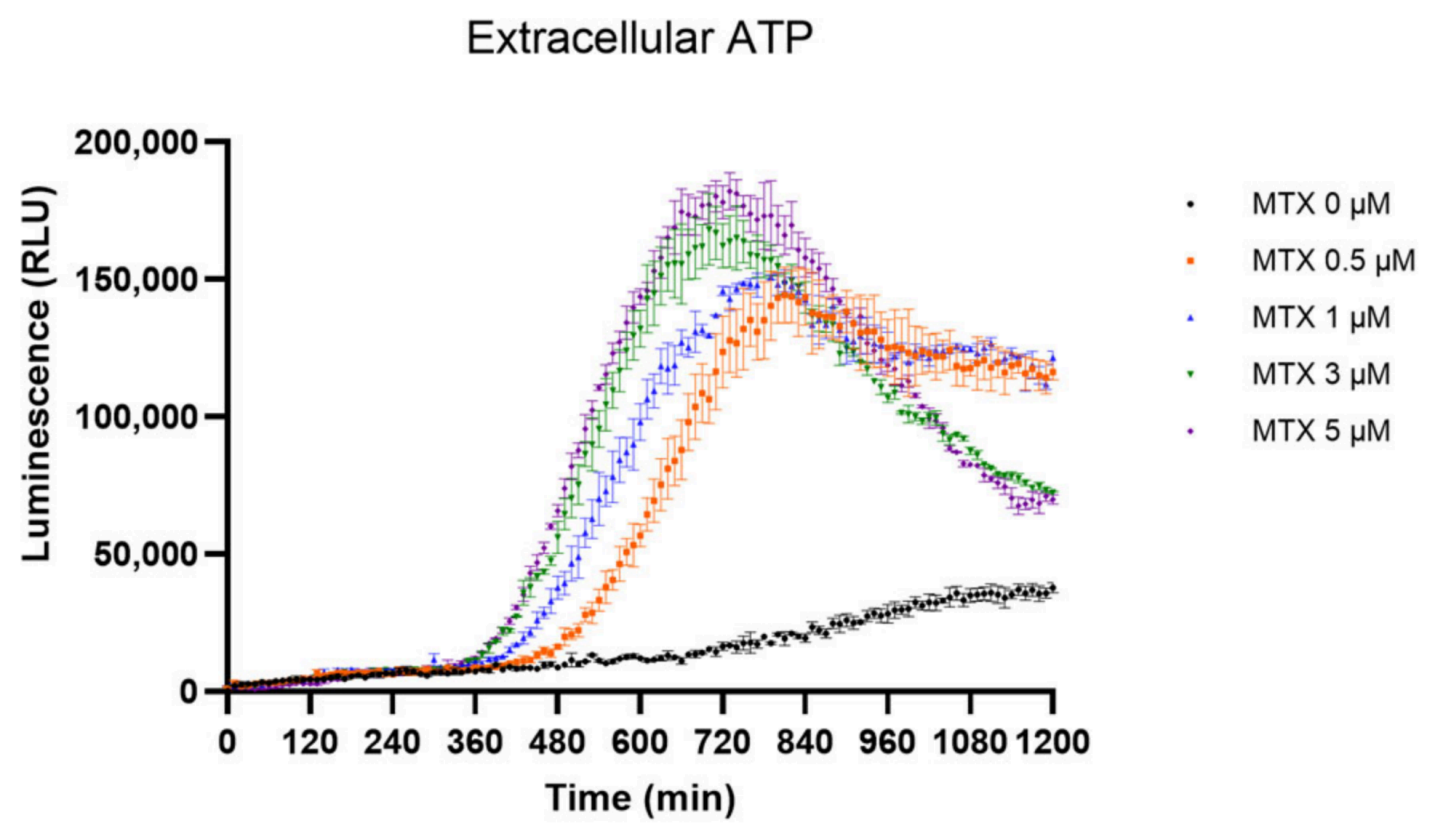
MTX induces the release of extracellular ATP in CT-26 cells. Extracellular ATP in CT-26 cultures exposed to MTX (0.5–5 μM) or vehicle, monitored continuously by a luminescence-based real-time assay over 20 h. Data are mean ± SEM from two independent experiments (*n* = 2).

We next examined extracellular ATP, a DAMP secreted during early apoptosis [24]. ATP was monitored continuously in CT-26 cultures exposed to MTX (0.5–5 μM) or vehicle using a luminescence-based real-time assay (Figure 3). Vehicle-treated cultures showed a slow, gradual rise in luminescence over the recording period. In MTX-treated cultures, ATP release began at approximately 360 min, and the extracellular ATP signal rose steeply thereafter at all concentrations, with kinetics differing across treatment conditions. ATP release occurred earlier at the higher concentrations (3 and 5 μM), which the ATP signal reaching a maximum sooner and then declining over the remainder of the recording, whereas at the lower concentrations (0.5 and 1 μM) release proceeded more gradually and was better sustained. The relative order of the groups therefore reversed over time, with the higher concentrations giving the greater signal early on and the lower concentrations doing so by the end of the recording. Together with the shift in the early-to-late apoptotic composition described above, these findings indicate that MTX concentration affected the kinetics and composition of the cellular response during the in vitro exposure period. We therefore used 1 and 3 μM, which showed distinct apoptotic profiles and ATP-release kinetics in the in vitro experiments.

### 2.4. Vaccination with MTX-Treated CT-26 Cells Protects Against Tumor Challenge

**Figure 4:**
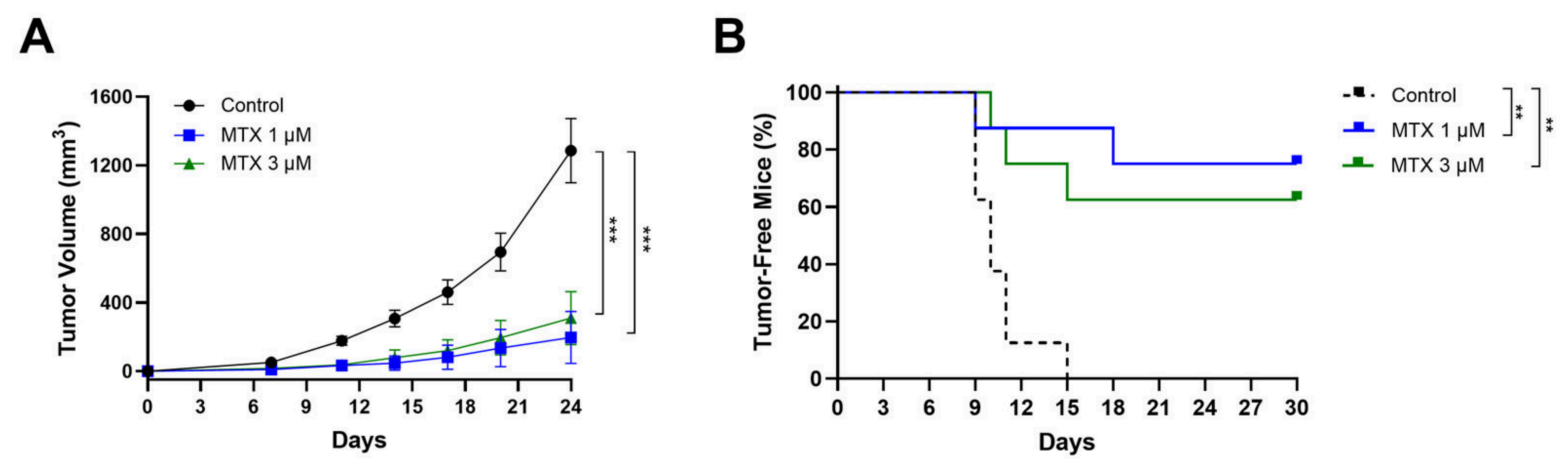
Prophylactic vaccination with MTX-treated CT-26 cells protects against tumor challenge. BALB/c mice were vaccinated subcutaneously with 1 × 10^6^ CT-26 cells treated with MTX (1 or 3 μM) for 24 h or with DPBS (control) and challenged 7 days later with 1 × 10^5^ live CT-26 cells in the contralateral flank. (**A**) Mean tumor volume at the challenge site. Data are mean ± SEM, *n* = 8 per group. Tumor volumes at day 24 were compared by one-way ANOVA with Dunnett’s multiple comparisons test. (**B**) Time to tumor onset, defined as the first of two consecutive measurements exceeding 100 mm^3^. Mice remaining tumor-free at day 30 were censored. Groups were compared by log-rank (Mantel–Cox) test with Bonferroni correction for two comparisons versus control. ** *p* < 0.01 and *** *p* < 0.001 versus control.

To test whether MTX-induced cell death is immunogenic, BALB/c mice were inoculated subcutaneously with 1 × 10^6^ CT-26 cells treated with MTX (1 or 3 μM) for 24 h, or with DPBS, and challenged 7 days later with 1 × 10^5^ live CT-26 cells in the contralateral flank. No tumor developed at any vaccination site. Control tumors grew progressively to approximately 1300 mm^3^ by day 24, whereas tumor volumes in both vaccinated groups remained below 350 mm^3^ (Figure 4A). Because most vaccinated animals never developed a tumor, these group means are dominated by the minority in which one arose, and incidence is therefore presented as time to tumor onset (Figure 4B). All control mice developed tumors within 15 days, whereas 6 of 8 (75.0%) and 5 of 8 (62.5%) mice vaccinated with cells treated at 1 and 3 μM, respectively, remained tumor-free.

### 2.5. Vaccination Efficacy Varies with the Interval Between Vaccination and Challenge

**Figure 5:**
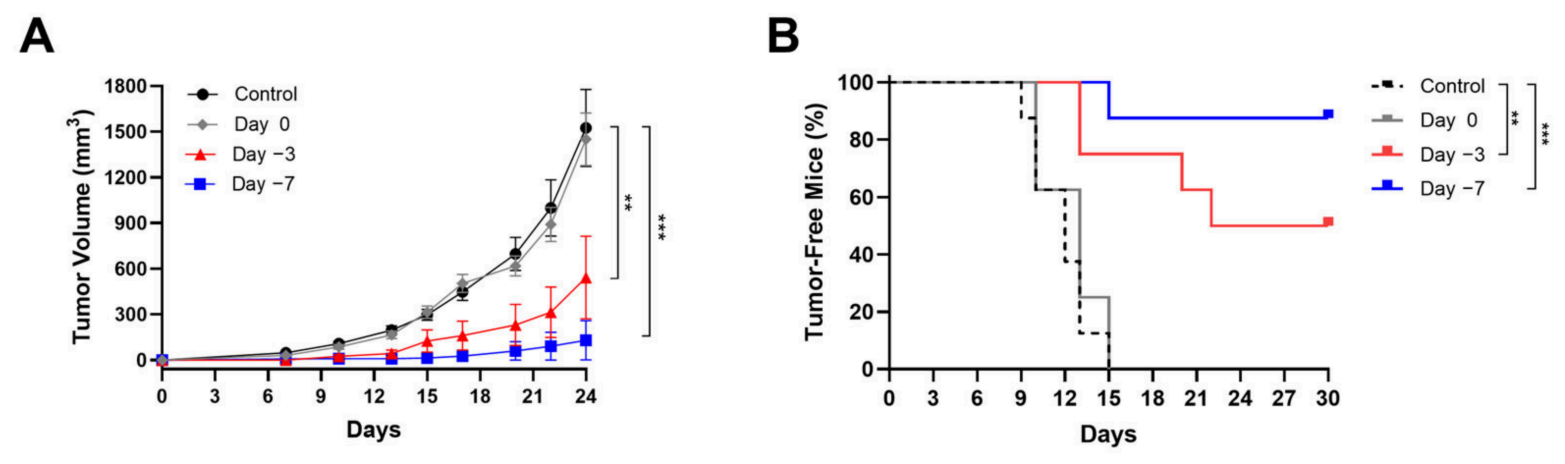
Vaccination efficacy varies with the interval between vaccination and challenge. BALB/c mice were vaccinated subcutaneously with 1 × 10^6^ CT-26 cells treated with MTX (1 μM) for 24 h on the day of challenge (Day 0) or 3 or 7 days beforehand (Day −3, Day −7) or received DPBS (control). All mice were challenged with 1 × 10^5^ live CT-26 cells in the contralateral flank on day 0. (**A**) Mean tumor volume at the challenge site. Data are mean ± SEM, *n* = 8 per group. Tumor volumes at day 24 were compared by one-way ANOVA with Dunnett’s multiple comparisons test. (**B**) Time to tumor onset, defined as the first of two consecutive measurements exceeding 100 mm^3^. Mice remaining tumor-free at day 30 were censored. Groups were compared by log-rank (Mantel–Cox) test with Bonferroni correction for three comparisons versus control. ** *p* < 0.01 and *** *p* < 0.001 versus control.

Because priming of naive T cells requires several days, we investigated whether the interval between vaccination and challenge determines the outcome. Mice were vaccinated with CT-26 cells treated with MTX (1 μM) for 24 h either on the day of challenge (Day 0) or 3 or 7 days beforehand (Day −3, Day −7). A separate control group received DPBS. As in the preceding experiment, no tumor developed at any vaccination site. Tumor growth in the Day 0 group was indistinguishable from that of the control group (Figure 5A), and all animals in both groups developed tumors within 15 days (Figure 5B). Because these mice received an identical inoculum, the lack of effect at Day 0 excludes a direct, non-immunological action of the injected cells on tumor engraftment. Protection was associated with a longer vaccination-to-challenge interval. Tumor volumes were reduced in the Day −3 group and further reduced in the Day −7 group (Figure 5A). At day 30, 4 of 8 mice (50.0%) vaccinated 3 days before the challenge and 7 of 8 (87.5%) vaccinated 7 days before the challenge remained tumor-free, compared with 0 of 8 in both the control and Day 0 groups (Figure 5B). Together with the absence of any effect at Day 0, this pattern is consistent with a requirement for a priming interval in which the outcome depends on a priming phase preceding tumor challenge rather than on the injected material itself.

### 2.6. Vaccination Efficacy Differs Among Cell Preparations Generated by Different Death-Inducing Conditions

**Figure 6:**
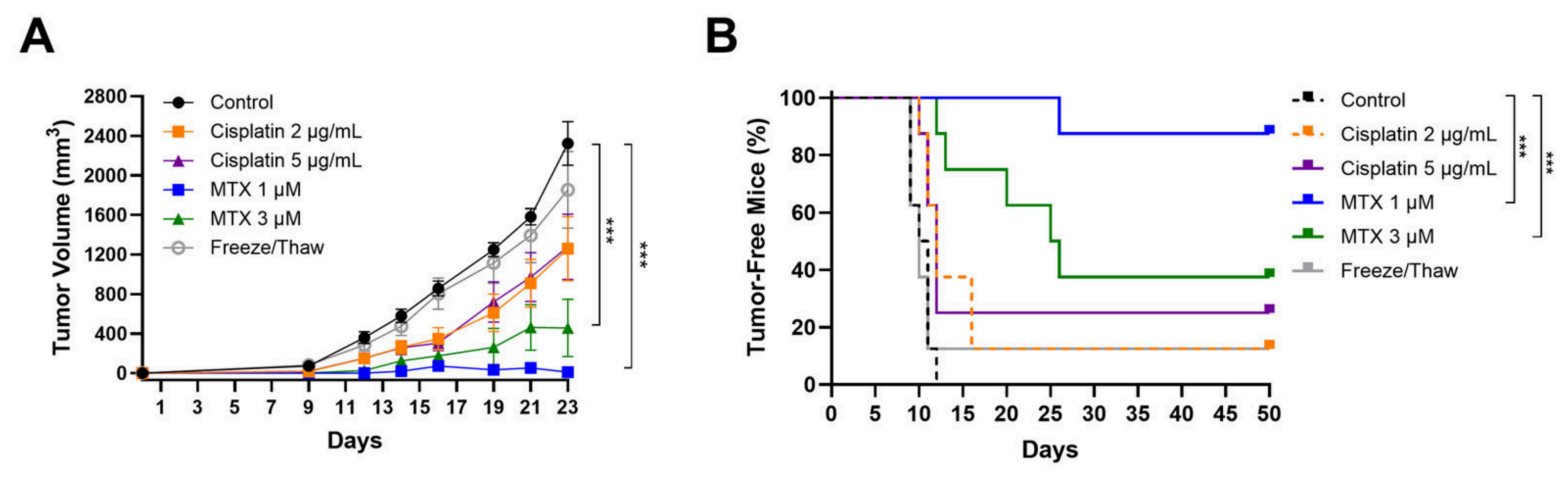
Vaccination efficacy differs according to the mode of cell death. BALB/c mice were vaccinated subcutaneously with 1 × 10^6^ CT-26 cells treated for 24 h with MTX (1 or 3 μM) or cisplatin (2 or 5 μg/mL), or subjected to four freeze–thaw cycles, or were given DPBS (control), and were challenged 7 days later with 1 × 10^5^ live CT-26 cells in the contralateral flank. (**A**) Mean tumor volume at the challenge site. Data are mean ± SEM, *n* = 8 per group. Mean values are shown up to day 23, the last time point at which all animals in every group remained in the study. Tumor volumes at day 23 were compared by one-way ANOVA with Dunnett’s multiple comparisons test. (**B**) Time to tumor onset, defined as the first of two consecutive measurements exceeding 100 mm^3^. Mice remaining tumor-free through day 50 were censored, and seven of eight mice in the 1 μM MTX group were subsequently used for the rechallenge experiment. Groups were compared by log-rank (Mantel–Cox) test with Bonferroni correction for five comparisons versus control. *** *p* < 0.001 versus control.

To compare the vaccination efficacy of cell preparations generated by different death-inducing conditions, we compared MTX with freeze–thaw (F/T), a canonical non-immunogenic form of accidental necrosis, and with cisplatin, a cytotoxic agent that does not engage the ICD program. Mice were vaccinated with CT-26 cells treated under each condition and challenged 7 days later. Similarly, no tumor developed at any vaccination site. Cells subjected to four freeze–thaw cycles were inactive as a vaccine. Tumor growth followed that of the control group (Figure 6A); only 1 of 8 mice remained tumor-free at day 50, and time to onset was indistinguishable from the control (Figure 6B). Cisplatin conferred only a marginal delay at either concentration (1 of 8 and 2 of 8 were tumor-free at 2 and 5 μg/mL; median onset was 12.0 days versus 10.5 days in controls). MTX was effective at both concentrations, with 7 of 8 mice (87.5%) tumor-free at 1 μM and 3 of 8 (37.5%) at 3 μM through day 50. Taken together, efficacy was not determined by the extent of cell death. Freeze–thaw rendered all cells non-viable yet conferred no benefit, whereas MTX-treated cells were markedly protective.

### 2.7. Vaccinated Mice Remain Resistant to Rechallenge

**Figure 7:**
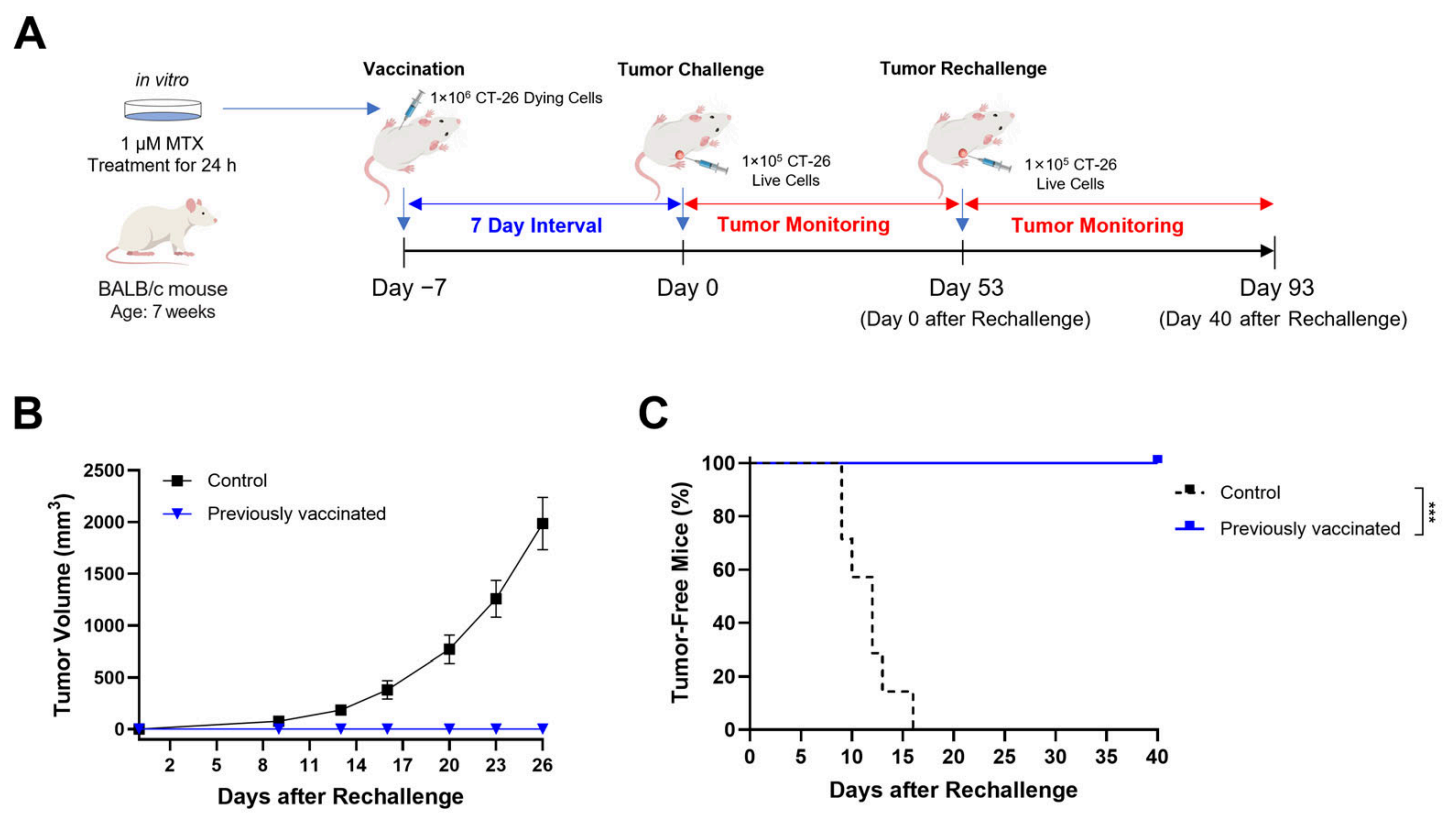
Vaccinated mice remain resistant to rechallenge. (**A**) Experimental scheme. BALB/c mice were vaccinated subcutaneously with 1 × 10^6^ CT-26 cells treated with MTX (1 μM) for 24 h on day −7 and inoculated with 1 × 10^5^ live CT-26 cells on day 0. The seven mice in the MTX (1 μM) group that remained tumor-free through day 50 were re-inoculated with 1 × 10^5^ live CT-26 cells on day 53, together with age-matched naive controls, and were monitored until day 93. (**B**) Mean tumor volume after rechallenge. Data are mean ± SEM, *n* = 7 per group. (**C**) Time to tumor onset after rechallenge, defined as the first of two consecutive measurements exceeding 100 mm^3^. Time zero corresponds to the day of re-inoculation. Mice remaining tumor-free through the end of the observation period were censored. Groups were compared by log-rank (Mantel–Cox) test. *** *p* < 0.001 versus control.

To determine whether the immunity established by vaccination was durable, the seven mice that had remained tumor-free through day 50 in the previous experiment following vaccination with MTX (1 μM)-treated cells were re-inoculated with 1 × 10^5^ live CT-26 cells on day 53, alongside age-matched naive mice (Figure 7A). All naive control mice developed progressively growing tumors, confirming the tumorigenicity of the inoculum, whereas tumor volumes in previously vaccinated mice remained at baseline throughout (Figure 7B). Tumor onset occurred within 16 days of re-inoculation in every control animal, whereas none of the previously vaccinated mice developed a palpable tumor at any point, and all remained tumor-free until the end of the observation period on day 93, corresponding to 40 days after rechallenge (Figure 7C). These results suggest that a single vaccination with MTX-treated cells establishes durable protection, sufficient to reject a second inoculum of live tumor cells administered without any further intervention.

## 3. Discussion

Using MTX as a reference ICD inducer, we varied the concentration used to generate the dying-cell vaccine and the interval between vaccination and challenge, two parameters that are usually held fixed. MTX-treated cells conferred marked and durable protection, and animals protected by vaccination resisted a subsequent rechallenge, but the outcome was not the same under all conditions. Neither freeze–thaw nor cisplatin reproduced the effect. Four freeze–thaw cycles rendered the entire preparation non-viable yet produced a time to tumor onset indistinguishable from that of untreated controls. Accidental necrosis of this kind releases intracellular contents without any preceding stress response, so that calreticulin is never translocated to the cell surface and the material is delivered to the immune system as debris rather than as a dying cell [4]. Cisplatin kills efficiently but does not trigger the PERK–eIF2α axis that drives pre-apoptotic calreticulin exposure, and its immunogenicity can be restored by supplying ER stress pharmacologically [16]. Consistent with this, it delayed tumor onset only marginally here. What determined the outcome was therefore not how many cells were killed but the cellular program through which death proceeded.

Vaccination on the day of challenge was entirely ineffective, whereas a 7-day interval left most animals tumor-free, with an intermediate outcome at 3 days. Because all groups received an identical inoculum, and because the vaccine and challenge were given in opposite flanks and therefore drained to different lymph nodes, the absence of any effect on the day of the challenge excludes a direct action of the injected cells on tumor engraftment and locates the difference in the timing of a systemic host response. Dendritic cells must first acquire antigen at the injection site, mature, and migrate to the draining lymph node [25], where naive CD8^+^ T cells are cross-primed by type 1 conventional dendritic cells, expand clonally, and differentiate into effectors before leaving the node, a sequence that occupies the better part of a week [26,27]. A vaccine given on the day of challenge cannot complete this sequence before the challenge inoculum establishes itself, whereas one given 7 days earlier can. The protection was also durable. Animals that rejected the first challenge remained tumor-free after a second inoculum given 53 days later, an interval long enough for the initial effector population to have declined, indicating that a single vaccination was sufficient to establish durable protective immunity. The vaccination-to-challenge interval is therefore not a procedural detail but an experimental variable capable of converting a protective preparation into an apparently inactive one, a principle that extends to the clinic, where the immunogenicity of therapeutic cancer vaccines has likewise been shown to depend on their timing relative to other systemic therapy [28].

Our in vitro data indicate where within the death program the relevant differences lie. At the time point used to prepare the vaccines, the total proportion of Annexin V-positive cells was similar across MTX concentrations, but its composition was not. Lower concentrations yielded a population dominated by early apoptotic cells, whereas late apoptotic cells predominated at higher concentrations. This distinction is immunologically consequential, because calreticulin is exposed on the cell surface during the pre-apoptotic phase and functions as an eat-me signal that facilitates uptake of dying cells by dendritic cells and subsequent antigen presentation [15,29]. Cells that have progressed to secondary necrosis have already discharged much of their soluble DAMP content, and the immunological output of such preparations differs qualitatively from that of intact apoptotic corpses [4]. The extracellular ATP measurements pointed in the same direction since release was faster but less sustained at the higher concentrations. Consequently, lower concentrations yielded a greater signal by the end of the recording. The transient ATP response at higher concentrations may reflect differences in the timing of ATP release and subsequent extracellular ATP turnover. Cells driven rapidly toward secondary necrosis exhaust their releasable ATP pool sooner, and extracellular ATP is continuously hydrolyzed by the ectonucleotidases CD39 and CD73 to immunosuppressive adenosine [30]. However, the mechanisms underlying the divergence in ATP kinetics were not directly examined in this study. What reaches a dendritic cell is therefore governed by the balance between release and degradation over time rather than by the peak concentration. The in vivo outcome was consistent with this interpretation. Cells treated at 1 μM were at least as effective as those treated at 3 μM in every experiment in which both were tested, despite comprising no greater proportion of dying cells. The concentration that produces the most cell death is therefore not necessarily the one that produces the most protection.

Our study has several limitations. Neither surface calreticulin exposure nor HMGB1 release was measured, and type I interferon signaling was not assessed, so the DAMP characterization was limited to extracellular ATP. Because ICD is defined functionally by the vaccination assay rather than by any individual marker [13,31], this does not affect the in vivo interpretation, but the difference between concentrations could not be attributed to a specific DAMP. We did not perform antigen-specific readouts or deplete immune cell subsets, so the effector requirements for the observed efficacy were not established directly. The concentration range was limited to two values selected around the IC_50_, and the difference between them did not reach statistical significance in the direct comparison, so we cannot define the boundaries of the effective window. In addition, Annexin V/7-AAD staining does not determine the long-term proliferative capacity of Annexin V-negative cells after MTX removal. Therefore, the Annexin V-negative population should not be interpreted as necessarily retaining normal proliferative viability. Finally, the prophylactic vaccination assay measures resistance to a subsequent challenge and does not directly predict therapeutic efficacy against established tumors. Further studies using additional ICD inducers, antigen-specific readouts, immune cell depletion, and a wider range of exposure conditions will be required to determine whether the findings extend beyond MTX, define which stage of the death process is optimal, and identify the effector populations mediating the observed protection.

Taken together with published studies, our results suggest that part of the variability of ICD vaccination experiments across laboratories may reflect differences in experimental conditions rather than in the agents themselves. We found here that an interval of zero days abolished the protection conferred by an otherwise effective preparation, and the same principle is evident with other ICD inducers. Photodynamic therapy in CT-26 is immunogenic at the IC_50_ and markedly less so at higher photosensitizer concentrations or light doses [19], and the immunogenicity reported for ferroptotic cells has differed between studies, a discrepancy that has been attributed to differences in how the dying cells were prepared rather than to the death pathway itself [10,11]. In each case, the agent is unchanged and only the conditions differ, so an agent tested once, at one concentration and one interval, may be classified as non-immunogenic when another set of conditions would have produced protection. Biological differences between cell lines, hosts, and death pathways remain important, but conditions cannot be assumed to be neutral. Because preparations described in identical terms may differ in the proportion of cells at each stage of death, reporting the composition of the inoculum at the time of injection, together with the vaccination-to-challenge interval and the outcome at the vaccination site, would make results comparable across laboratories.

## 4. Materials and Methods

### 4.1. Chemicals and Reagents

Mitoxantrone dihydrochloride (Cat no. M6545) and cisplatin (Cat no. P4394) were purchased from Sigma-Aldrich (St. Louis, MO, USA). MTX was dissolved in DMSO and cisplatin in DPBS, and working solutions were prepared in culture medium before use. Roswell Park Memorial Institute (RPMI) 1640 medium, fetal bovine serum (FBS), Dulbecco’s phosphate-buffered saline (DPBS), trypsin-EDTA solution, and penicillin/streptomycin were purchased from Gibco (Grand Island, NY, USA). Annexin V-FITC and 7-AAD were purchased from Biolegend (San Diego, CA, USA). Matrigel was purchased from Corning, Inc. (Corning, NY, USA).

### 4.2. Cell Lines and Culture

The murine colon carcinoma line CT-26 (BALB/c origin) was obtained from the Korean Cell Line Bank (Seoul, Korea) and maintained in RPMI-1640 supplemented with 10% FBS, 100 units/mL penicillin, and 100 μg/mL streptomycin at 37 °C in a humidified atmosphere containing 5% CO_2_. The medium was replaced every 2–3 days, and cells were subcultured at 80–90% confluence using 0.25% trypsin-EDTA.

### 4.3. Determination of Half-Maximal Inhibitory Concentrations

CT-26 cells were seeded in 96-well plates at 1 × 10^4^ cells per well and allowed to adhere overnight. The medium was then replaced with 100 μL of medium containing MTX (0.05–10 μM) or vehicle, and cells were incubated for 24 h. Viability was assessed using the MTS assay and the CellTiter-Glo Luminescent Cell Viability Assay (Promega, Madison, WI, USA) according to the manufacturers’ instructions. Viability was expressed relative to vehicle-treated controls. IC_50_ values were determined by nonlinear regression using a four-parameter logistic model with the top and bottom constrained to 100% and 0%, respectively, in GraphPad Prism 10 (GraphPad Software, San Diego, CA, USA).

### 4.4. Analysis of Apoptosis

CT-26 cells were seeded in 6-well plates at 5 × 10^5^ cells per well and incubated overnight. Cells were treated with MTX (0.2–5 μM) or vehicle for 6, 12, or 24 h. Following treatment, both adherent and floating cells were collected and combined. Cells were washed in cold DPBS, resuspended in Annexin V binding buffer, and stained with FITC-conjugated Annexin V and 7-AAD for 15 min at room temperature in the dark. Populations were defined as viable (Annexin V^−^/7-AAD^−^), early apoptotic (Annexin V^+^/7-AAD^−^), late apoptotic (Annexin V^+^/7-AAD^+^), and Annexin V^−^/7-AAD^+^. Annexin V-FITC was detected in the FITC channel and 7-AAD in the PerCP-Cy5.5 channel. At least 20,000 events per sample were acquired on a BD LSRFortessa X-20 flow cytometer (BD Biosciences, San Diego, CA, USA) and analyzed with FlowJo 10.0 (BD Biosciences). The Annexin V^−^/7-AAD^+^ population was detected at negligible levels under the experimental conditions.

### 4.5. Measurement of Extracellular ATP

Real-time extracellular ATP accumulation was monitored with the RealTime-Glo Extracellular ATP Assay (Promega). CT-26 cells were plated at 1 × 10^4^ cells per well in white 96-well plates in L-15 medium containing 10% FBS, 100 units/mL penicillin, and 100 μg/mL streptomycin. Following the addition of MTX (0.5–5 μM) or vehicle, the detection reagent was dispensed into each well, and luminescence was recorded at 10 min intervals for 20 h at 37 °C using a SpectraMax i3 microplate reader (Molecular Devices, San Jose, CA, USA). Wells containing medium and drug but no cells were included at every concentration to correct for background signal.

### 4.6. Animals

Six-week-old male BALB/c mice were purchased from Saeron Bio (Uiwang, Republic of Korea) and housed under specific pathogen-free conditions with a 12 h light/dark cycle and ad libitum access to standard chow and water. Animals were acclimatized for at least 1 week before experimentation and randomly allocated to experimental groups. All procedures were approved by the Institutional Animal Care and Use Committee of the Korea Institute of Oriental Medicine (approval no. 24-042 on 26 April 2024, and no. 25-009 on 20 January 2025). Tumor size and animal condition were monitored two to three times per week. Animals were euthanized when the tumor burden reached 4000 mm^3^ or when humane endpoints were met, including tumor ulceration or marked deterioration in general condition. Euthanasia was performed by CO_2_ inhalation.

### 4.7. Preparation of Dying Cells and Prophylactic Vaccination

CT-26 cells were seeded at 5 × 10^5^ cells per well in 6-well plates and treated for 24 h with MTX (1 or 3 μM) or cisplatin (2 or 5 μg/mL). For freeze–thaw treatment, cells were subjected to four cycles of freezing at −80 °C and thawing at 37 °C. Adherent and floating cells were pooled, washed three times in DPBS, and resuspended in DPBS at 1 × 10^7^ cells/mL. An aliquot corresponding to 1 × 10^6^ cells was used for each vaccination. Mice received a subcutaneous injection of 1 × 10^6^ treated cells in 100 μL DPBS into the left flank. Control mice received an equal volume of DPBS. Unless otherwise stated, mice were challenged 7 days later with 1 × 10^5^ live CT-26 cells in 100 μL of DPBS containing 30% Matrigel injected subcutaneously into the contralateral flank. For the interval experiment, vaccination was performed 7 or 3 days before challenge, or on the day of challenge. Vaccination sites were inspected at each tumor measurement for local tumor growth.

### 4.8. Tumor Monitoring and Rechallenge

Tumor growth at the challenge site was monitored two to three times per week with digital calipers, and tumor volume was calculated as (length × width^2^)/2. Time to tumor onset was defined as the first of two consecutive measurements exceeding 100 mm^3^. Animals in which this criterion was not reached by the end of the observation period are referred to as tumor-free. Tumor volume curves are shown up to the last time point at which all animals in every group remained in the study. Mice that had not developed a tumor by that time were followed thereafter, and the complete observation period is presented as time to tumor onset. For rechallenge, the seven mice that had remained tumor-free through day 50 after vaccination with MTX (1 μM)-treated cells in the primary experiment were re-inoculated with 1 × 10^5^ live CT-26 cells on day 53, together with age-matched naive BALB/c mice, and were monitored until day 93.

### 4.9. Statistical Analysis

Data are presented as mean ± SD or mean ± SEM as indicated in the figure legends. Tumor volumes were compared by one-way ANOVA with Dunnett’s multiple comparisons test against the control group. Time to tumor onset was estimated by the Kaplan–Meier method, with the day of challenge as time zero, or the day of re-inoculation for the rechallenge experiment. Groups were compared by the log-rank (Mantel–Cox) test with Bonferroni correction for the number of comparisons against the control group. Mice remaining tumor-free at the end of the observation period were censored. All analyses were performed in GraphPad Prism 10, and *p* < 0.05 was considered statistically significant.

## 5. Conclusions

MTX-treated CT-26 cells conferred marked and durable protection as a prophylactic vaccine in immunocompetent mice, and the outcome depended on conditions that are usually held fixed. Protection was abolished when vaccination and challenge were performed on the same day and was maximal at an interval of 7 days, and neither freeze–thaw nor cisplatin reproduced the effect despite killing cells efficiently. At the exposure time used to prepare the vaccines, the total extent of cell death was similar across MTX concentrations, while late apoptotic cells came to predominate at higher concentrations. The identity of an ICD-inducing agent therefore does not by itself determine whether a dying-cell vaccine will protect, and the conditions under which it is prepared and administered should be reported.

## Data Availability

The original data presented in this research are included in the article. Further inquiries can be directed to the corresponding author.
